# Using a Scenario-Based Approach to Teaching Professionalism to Medical Students: Course Description and Evaluation

**DOI:** 10.2196/26667

**Published:** 2021-06-24

**Authors:** James Ashcroft, Patrick Warren, Thomas Weatherby, Stephen Barclay, Laurence Kemp, Richard Justin Davies, Catherine Elizabeth Hook, Elizabeth Fistein, Elizabeth Soilleux

**Affiliations:** 1 University of Cambridge Cambridge United Kingdom

**Keywords:** medical education, curriculum, training, professionalism

## Abstract

**Background:**

Doctors play a key role in individuals’ lives undergoing a holistic integration into local communities. To maintain public trust, it is essential that professional values are upheld by both doctors and medical students. We aimed to ensure that students appreciated these professional obligations during the 3-year science-based, preclinical course with limited patient contact.

**Objective:**

We developed a short scenario-based approach to teaching professionalism to first-year students undertaking a medical course with a 3-year science-based, preclinical component. We aimed to evaluate, both quantitatively and qualitatively, student perceptions of the experience and impact of the course.

**Methods:**

An interactive professionalism course entitled Entry to the Profession was designed for preclinical first-year medical students. Two scenario-based sessions were created and evaluated using established professionalism guidance and expert consensus. Quantitative and qualitative feedback on course implementation and development of professionalism were gathered using Likert-type 5-point scales and debrief following course completion.

**Results:**

A total of 70 students completed the Entry to the Profession course over a 2-year period. Feedback regarding session materials and logistics ranged from 4.16 (SD 0.93; appropriateness of scenarios) to 4.66 (SD 0.61; environment of sessions). Feedback pertaining to professionalism knowledge and behaviors ranged from 3.11 (SD 0.99; need for professionalism) to 4.78 (SD 0.42; relevance of professionalism). Qualitative feedback revealed that a small group format in a relaxed, open environment facilitated discussion of the major concepts of professionalism.

**Conclusions:**

Entry to the Profession employed an innovative approach to introducing first-year medical students to complex professionalism concepts. Future longitudinal investigations should aim to explore its impact at various stages of preclinical, clinical, and postgraduate training.

## Introduction

The medical profession is an ancient profession, whose members are regarded as important and respected members of society. Doctors play a key role in many aspects of individuals’ lives, with a more holistic integration into society than simply as those who can diagnose and treat disease. Individuals entrust doctors with information that they might tell no one else and are prepared, as relevant, to be subjected to intimate examinations by them. It follows that the doctor must show exemplary conduct to justify this level of trust and respect. Because public trust in doctors and the regulation and accountability of the profession is vital for the effective practice of medicine, core professional values must be upheld, not only by those who are qualified, but also by medical students. Unprofessional behavior at medical school is associated with early academic difficulties [1], unsatisfactory progress [2], and poor clinical performance [3] and predicts subsequent serious misconduct among practitioners [4].

The UK General Medical Council, Medical Schools Council, and British Medical Association have provided relatively extensive guidance about the responsibilities of medical students with respect to professional behavior [5]. Much of what is formally taught in medicine is about the knowledge, skills, and behaviors required of a physician, including how to express compassion and respect for patients at the bedside [6]. Every year, concern is raised about the conduct of medical students, with a small number refused provisional registration to practice [7]. In 2016, 13% of applications for provisional registration with the General Medical Council from final year medical students included declarations about fitness to practice issues, although the majority of applications were ultimately successful [8].

Given the potentially profound implications of professional misconduct, a proactive approach from the beginning of the medical course might be preferable over waiting to redress problems that have already occurred. Formal methods have previously been considered for the delivery of the professionalism curriculum. In the traditional view of medicine, there is a dominant testing culture heavily influenced by behaviorist learning theory, belief in objective and standardized testing, and separation of testing from instruction [9,10]. This perspective has allowed conceptualization of professionalism as correct or desirable character traits or behaviors and has been useful in developing summative tools for assessment of progression. A recent shift toward a constructivist learning paradigm based on intersubjectivity instead of classical objectivity accepts that professionalism is not a stable construct that can be isolated, taught, and assessed but a set of sophisticated and socially constructed competencies that can be taught and refined over a lifetime [11,12]. An extension of this concept is the development of vignettes or scenarios to enable reflection on realities of professionalism as enacted in practice. Several studies have successfully used standardized professional dilemmas to explore how students conceptualize appropriate or inappropriate behavior and how they come to decisions about how they might act [13-17]. Vignettes or scenarios relating to professionalism have also been used very successfully for postgraduate clinical trainees in ophthalmology [18] and pathology [19]. However, no previous studies have undertaken early professionalism in undergraduate medical students prior to clinical experience, which is the case for a traditional preclinical medical school curriculum in the United Kingdom. Traditional preclinical medical students here undertake a scientific preclinical course of study involving minimal patient contact or clinical experience, and they are surrounded by students studying a wide range of other subjects. In many ways, this is a positive aspect of their student experience, but there is a risk that students do not identify as future medical professionals and so become involved in behaviors at odds with their fitness to practice obligations.

Applying the aforementioned principles has allowed for the development of a short course described in this report with the aim of ensuring that all first-year preclinical medical students understand their obligations with respect to professional conduct. This is the first report of professionalism training in this unique cohort preceding all clinical patient interaction.

## Methods

### Design and Setting

A session-based short course was developed and incorporated into the first year of undergraduate medical training at the School of Clinical Medicine, University of Cambridge, United Kingdom. Key areas of concern for student fitness to practice were identified through adaptation of previously published professional competences defined in medical practice [20,21], guidance from the General Medical Council on professional behavior and fitness to practice [5], and expert opinion from clinical educators and mentors.

Two 90-minute sessions each dealing with 3 areas of concern were designed by experienced clinical educators working with senior medical students with experience in a range of clinical environments to increase both authenticity and peer impact (Textbox 1).

**Entry to the profession: facilitator’s guide.**

Learning objectives

- By the end of this activity, learners will be able to perform the following:
  Describe fundamental principles of medical professionalism
  Apply standards of professionalism to their day-to-day lives as undergraduate medical students
  Begin to recognize professionalism problems that could arise in a medical setting

Introduction

- Learners are informed about the professionalism training and give informed consent to participate

Initial meeting

- Course structure is explained, and participants are introduced to facilitators and individual groups

Room plan

- Each session takes place in an open university room allowing for engagement within a circle seating arrangement

Session 1 topics

- Interacting with professional colleagues (issues: academic bullying, confidentiality, whistle blowing, public confidence in the profession)
- Respecting colleagues (issues: lying, respect for colleagues, racism, sexism, competence/patient safety, team working)
- Maintaining professional behavior in all aspects of life (issues: alcohol, lying, respect for colleagues, racism, competence/patient safety, sexual consent)

Session 2 topics

- Health and probity (issues: alcohol/addiction, lying, mental health)
- Photos and communication (issues: confidentiality and probity, respect for colleagues and patients, consent for use of publication of photographic material)
- Presentation and conduct (issues: appearance, sphere of competence, data protection)

Debrief

- Participants undertake debrief conducted by facilitators of varying experiences that encourages reflection on the conversation topics

Participant evaluation

- Postsimulation questionnaire and open-ended interview explore participant experiences

Each area of concern was introduced with a hard-hitting scenario involving a qualified doctor doing something that is obviously wrong. Following this introductory scenario, 3 more nuanced scenarios were presented probing related topics. These additional scenarios were more pertinent to medical students and facilitated more thoughtful discussion than a simple comment that what was occurring was clearly inappropriate. In total, each session comprised 12 scenarios divided into 4 groups, each thematically associated with one of the introductory scenarios relating to qualified doctors. To ensure active or experiential learning, students were provided with scenarios/vignettes to read prior to each session (Multimedia Appendix 1). Discussions were centered on judgments about whether conduct was appropriate or inappropriate and how the individuals depicted in the scenarios might have behaved more professionally.

### Participants

A total of 70 first-year medical students attended the short course as a mandatory component of their undergraduate course. In order to maximize participation within groups, students were divided into groups of 3 to 4 with each group being guided by a facilitator. Clinical facilitators from medical, surgical, clinical laboratory, and primary care specialties with varying backgrounds and senior clinical medical students were recruited and trained in facilitating the course and debriefing. We sought to maximize student perceptions of the applicability of professionalism to all aspects of their lives and not just those associated with lectures, practical classes, small group teaching sessions, and other aspects of academic learning. Accordingly, the sessions were contextualized by running them in an informal and nonthreatening evening setting with refreshments being included (Multimedia Appendix 2). Facilitators could read the scenarios in advance and discuss them with the course lead, who could direct them to relevant documents, particularly the General Medical Council, British Medical Association, and Medical Defense Union publications referred to in this article [5,7,22]. Facilitators were debriefed at the end of the session and could make suggestions for improving the material and raise any concerns about the course material or the attitude of any particular student.

### Data Collection

Small focus groups have been demonstrated to stimulate debate and insightful thoughts and encourage interactions key for data collection [23]. Anonymous feedback was collected from students in a quantitative and qualitative approach to gain insights into the course implementation and development of professionalism rather than assessing knowledge and/or understanding of issues related to professionalism. Quantitative data were gathered from students using a Likert-type 5-point scale (1=strongly disagree, 2=agree, 3=neutral, 4=agree, 5=strongly agree) on (1) acceptability of the format and subject matter of the course, (2) attitude toward professionalism before and after the course, (3) course delivery and perception of the course’s effect on their attitudes, (4) extent to which they believe issues of professionalism apply to them as students, and (5) perception of their own level of understanding of professionalism. Qualitative data was gathered through debrief feedback and white space questions following course completion. Data were captured on a secure web-based Google form accessible only to the program directors. No identifiable or personal health information was collected, and the course was not an educational experiment upon the students, so no ethical review was required.

### Data Analysis

Survey data analyses were undertaken for curriculum implementation and development of professionalism. Quantitative analysis of ordinal 5-point Likert-type responses was undertaken using SPSS (version 23.0, IBM Corp). Qualitative thematic analysis was undertaken using a 6-phase approach of familiarization including numbering of student responses, generating codes, searching for themes, reviewing themes, defining themes, and producing a report [24]. The coding scheme for qualitative analysis is demonstrated in Table 1. Student statements were categorized and rated independently by 2 authors (PW and JA) in terms of how positive or negative the statement was perceived on a scale of –2 to +2, allowing a mean score per category to be generated. The mean rating calculated for each category was multiplied by the total number of students who commented on the subcategory to provide semiquantitative analysis of total effect.

**Table 1:** Coding scheme for qualitative analysis of curriculum implementation and development of professionalism [24].

Category label			Criteria
**Curriculum implementation**			
	Course facilitators	Student refers to facilitators delivering course.	
	Environment	Student refers to atmosphere/environment of course.	
	Timing	Student refers to timing of course.	
	Quantity	Student refers to quantity of content used in course.	
	Quality of content	Student refers to content used in course.	
**Development of professionalism**			
	Challenge	Student refers to ease or difficulty of professionalism concepts covered in course.	
	Learning	Student refers to learning about professionalism concepts covered in course.	
	Revision	Student refers to revision of professionalism concepts covered in course.	
	Enjoyable	Student refers to enjoyment of course.	
	Engagement	Student refers to engagement in course.	

## Results

In the first year (2017-2018), 50 students participated in the session, and feedback and debriefs regarding course implementation were collected. Specific suggestions from student and facilitator feedback were considered and implemented as necessary. In the second year (2018-2019), 100 students participated in the course and were assessed in their development of professionalism. Within the 2 cohorts, 15 pilot participants and 55 subsequent participants who undertook 2 professionalism sessions volunteered quantitative feedback on the course. Following the second year of course implementation, 18 students volunteered to provide qualitative feedback regarding curriculum implementation and development of professionalism at the end of the course. The session leads collected feedback from students, took debriefs from facilitators, and wrote a brief contemporaneous record of all feedback to aid in implementing suggested changes in the next iteration. All 70 of the participants who gave feedback answered questions about their views on the practicalities and implementation of this professionalism curriculum by answering a quantitative postcourse survey (Table 2).

**Table 2:** Quantitative curriculum implementation feedback scores (n=70).

Curriculum implementation feedback	Value, mean (SD)
Environment in which the sessions were delivered allowed me to feel comfortable in sharing my honest opinions and asking questions.	4.66 (0.61)
Structure of the discussion was well designed and effective for achieving the aims of the session.	4.43 (0.69)
I now have a better understanding of what may be considered unprofessional behavior.	4.39 (0.79)
These sessions have improved my understanding and awareness of how issues surrounding professionalism affect me as a medical student.	4.37 (0.76)
I now feel more able to act appropriately if an event occurs that could potentially bring my or a friend or colleague’s professionalism into question.	4.29 (0.84)
Content of the scenarios and discussions was effective and covered most areas of professionalism that could affect me as a preclinical student.	4.23 (0.78)
I found these sessions useful and worthwhile to me as a medical student.	4.19 (0.95)
Scenarios and discussions were appropriate to me as a first-year medical student.	4.16 (0.93)

Mean scores on a Likert-type 5-point scale ranged from 4.16 (SD 0.93; appropriateness of scenarios) to 4.66 (SD 0.61; environment of sessions), indicating positive postcourse feedback. Semiquantitative participant feedback was gathered using a quantitative postcourse survey (Table 3).

**Table 3:** Quantitative development of professionalism feedback scores (n=70).

Development of professionalism feedback	Value, mean (SD)
Medical students should be expected to behave professionally.	4.78 (0.42)
I understand what is meant by professionalism.	4.71 (0.46)
Professionalism is a relevant topic for medical students in preclinical years.	4.62 (0.59)
I feel I can recognize professional and unprofessional behavior in my teachers.	4.04 (0.74)
My behavior in my preclinical medical studies is social and shouldn’t be evaluated.	3.87 (0.79)
Higher standards of professionalism are needed in preclinical medical education.	3.11 (0.99)

Mean scores on a Likert-type 5-point scale ranged from 3.11 (SD 0.99; need for professionalism) to 4.78 (SD 0.42; relevance of professionalism), indicating positive postcourse professionalism development. A total of 18 students engaged in qualitative feedback in curriculum implementation and development of professionalism as displayed in Table 4. The most frequent qualitative theme addressed was the quality of the sessions, with a strongly positive effect indicating that the sessions were well received by the students. Students were less likely to comment on the impact of the professionalism course on their learning or revision. Overall, feedback across all aspects of the sessions was positive with no significant concerns regarding course content or execution.

**Table 4:** Qualitative curriculum implementation and development of professionalism feedback.

Category			Value, mean (SD)		Number of contents		Total effect (mean × n)		Sample paraphrased comments from students (assigned comment number)
**Curriculum implementation**									
	Timing	–0.17 (0.41)		6.00		–1.00		There could be greater flexibility in the timing of the course (1, 6, 7, 9, 14, 18).	
	Quality of content	1.22 (0.97)		9.00		11.00		I thought the scenarios discussed were very useful and definitely helped put ideas that we may have already been aware of into practice (1, 2, 4, 6, 8, 10, 15, 14, 18).	
	Environment	2.00 (0)		5.00		10.00		The setting for the seminars (relaxed, with drinks and snacks, etc) created a friendly engaging atmosphere (3, 9, 12, 16, 18).	
	Course facilitators	1.60 (0.89)		5.00		8.00		Really valuable to have a current clinical student present (5, 7, 8, 9, 17).	
	Quantity	0.33 (0.58)		3.00		1.00		Maybe slightly reduce the number of cases presented (11, 14, 18).	
**Development of professionalism**									
	Challenge	0.33 (0.58)		3.00		1.00		The scenarios were sometimes quite obvious (1, 2, 6).	
	Revision	2 (0)		2.00		4.00		Definitely helped put ideas that we may have already been aware of into practice (10, 11).	
	Enjoyment	2 (0)		2.00		4.00		Loved the sessions (12, 16).	
	Learning	2.25 (0.50)		2.00		4.50		I now feel I have a much broader understanding of the levels of professionalism required as both a medical student and a doctor (17, 18).	
	Engagement	1.00 (0.89)		3.00		3.00		I felt that the open table group discussion was a bit intimidating simply because I am quieter than a lot of my peers (5, 10, 14).	

## Discussion

### Principal Findings

This approach to teaching professionalism was designed to introduce first-year preclinical medical students, undertaking a course with very limited patient contact in the first 3 years, to complex concepts relevant to medical students and qualified doctors in two 90-minute sessions. Both quantitative and qualitative feedback indicate that the sessions were very well received by students. Although still strongly positive, the least well-received themes revolved around the usefulness and appropriateness of the course content, with qualitative feedback revealing that this may be due to the challenge of the scenarios being too easy. Interestingly, the response to the question of whether higher standards of professionalism are required in the medical field was neutral. One reason may be that the very strong focus on preclinical science in the undergraduate UK medical course means that students have, at that stage, devoted little time to consideration of the meaning of professional conduct as applied to interactions with patients and colleagues. It is also difficult to judge how complex professionalism scenarios for first-year medical students should be, as there may be very significant variation in students’ prior experience in relevant professional situations. However, it is clear that professionalism is required for all practicing clinicians, and longitudinal studies of professionalism in medical students may give more insights into appropriate professionalism scenario complexity at this stage of training.

Quantitative and qualitative course feedback revealed that quality of content, environment, and course facilitators were the most positive factors overall. In this case, professionalism training was applied within the student collegiate system, where the majority of pastoral and small group teaching components of the medical course are delivered, thereby enhancing the familiarity of the group session. Using small group discussions with familiar college mentors and senior students as facilitators encouraged active engagement of students with the scenarios, which maximized their understanding and retention of the material. Feedback provided insights into less positively received timing and quantity aspects of the course, with students feeling the course at times tried to cover complex scenarios over short durations. Educational programs in an ideal world should be flexible, with differences between individual learners being identified and suitable learning tasks selected, therefore allowing all abilities of student to comfortably advance through course content [25]. To further improve this program, a greater emphasis could be placed on instructional design and stimulating the recall of prerequisite knowledge to allow a more seamless progression through scenarios [25,26]. Students felt that the course should restrict itself to covering fewer scenarios in order to fully explore professionalism dilemmas. This is understandable, as professional practice involves practitioners finding not so much the right answer (which may not always exist in any absolute sense) but rather in deciding what is best in the situation in which they find themselves [27]. The demanding medical curriculum, unfortunately, did not allow extension of the length of this professionalism course. Due to the complexity of professionalism, future iterations of the course will aim to provide more time for each scenario discussion to improve both engagement and learning.

### Limitations

Although this study collected a range of constructive feedback to enhance the provision and content of the course, students gave this feedback on a voluntary basis, and only 30% of the 2017-2018 cohort and 55% of the 2018-2019 cohort provided feedback. There is a risk that this induced selection bias, with students with either more positive or more extreme opinions preferentially providing feedback. All feedback was anonymized in order to minimize any perceived pressure to provide positive feedback. A further limitation of this study is its inability to precisely determine the effects of our professionalism intervention on the students’ subsequent clinical practice. The benefits of professionalism training might not be felt until after qualification as a doctor, which would need both detailed ethical approval and extensive follow-up in order to produce meaningful results. Unfortunately, long-term follow-up is difficult in medical students and medical doctors, as they frequently move between jobs due to the nature of training rotations and sometimes move in and out of research and/or other career breaks from medicine, with some leaving the country either temporarily or permanently.

### Conclusion

This approach to teaching professionalism is both interactive and experiential in nature and aimed at medical students in the first year of a traditional medical course. In particular, Entry to the Profession benefitted from a small group format in a relaxed and open environment with welcoming facilitators to successfully teach the major concepts of professionalism. Our study would benefit from a future longitudinal complementary investigation to explore the impact of medical student professionalism education at various stages of preclinical, clinical, and postgraduate training.

## Appendix

Multimedia Appendix 1 A scenario-based approach to teaching professionalism to medical students: case vignettes.

Multimedia Appendix 2 A scenario-based approach to teaching professionalism to medical students: session setup.
