# Supplementary material for: Using a Scenario-Based Approach to Teaching Professionalism to Medical Students: Course Description and Evaluation
Source: JMIR Med Educ. 2021 Jun 24;7(2):e26667. doi: 10.2196/26667 (PMC8277325; doi:10.2196/26667)
Supplement: Multimedia Appendix 1 [file mededu_v7i2e26667_app1.docx]

**1) Interactions with professional colleagues (issues: academic bullying, confidentiality, whistle blowing, public confidence in the profession)**

# CLINICAL PRACTICE SCENARIO

A senior male maxillofacial surgeon is supervising a middle grade trainee undertaking a complex jaw cancer resection and the junior doctor is struggling. A small artery is inadvertently cut through and the senior attending loses his temper, swearing at the junior doctor and telling him he is fat, undereducated and a disgrace to the profession. Another more junior attending hears the shouting from outside the operating theatre, but feels unable to inform anyone, because the senior attending is in charge of training junior doctors locally in Maxillofacial Surgery and he is concerned about his own professional future in the department if he “rocks the boat”.

*Are all of these doctors behaving in an appropriate way and, if not, what should they do differently*?

# RELATED SCENARIOS

1. As the weeks pass by in your first term, you notice that one of the students in your anatomy group seems to always be slightly behind the rest of the group on their anatomical knowledge; the rest of the group make light fun of this, always asking the student supposedly easy questions and laughing when they don’t know the answer. The student tries to laugh along too, but you can tell that they aren’t entirely comfortable with the ‘banter’. In one session, you jokingly ask if the student knows the difference between the trachea and the aorta; however, they burst into tears and leave the session.

1. Near the end of term, you observe a student in another dissection group discreetly taking a selfie on their phone with their donor’s heart in their hand. You are aware that this is against both dissection room rules and the law, but don’t want to be a snitch, and you also reason that a photograph of a heart does not contain identifiable information about the donor.

1. Whilst shopping at the weekend with some of your medic friends, the discussion turns to their experience of dissecting. Among the topics they discuss are some interesting aspects of their donors’ medical histories, the time one of them poked another student from behind with the donor’s hand, and which demonstrator is most attractive. You’re slightly reluctant to join in this conversation as it’s in such a public space.

*What is the line between banter and bullying? How can you help colleagues who are having difficulties academically?*

*Should you report this to the dissection room staff, or let it go? What is the potential impact of this; what if the photo were publicly shared on social media? How could it impact: the student; the dissection room; the public’s confidence in the profession?*

*Is this an acceptable conversation to have in a shop? How about over dinner at a restaurant? Or with family/friends on the phone?*

# 2) Respecting colleagues (issues: lying, respect for colleagues, racism, sexism, competence/patient safety, sexual consent, team working)

**CLINICAL PRACTICE SCENARIO**

A junior doctor writes a comment on his Facebook page after a tough week of night shifts and it leads to the following discussion:

Doctor A: “Another week of night shifts and stinking demented patients that don’t understand what darkness means!!”

Doctor B: “Maybe we should stop curing cancer, so people don’t end up like this!”

Doctor A: “Main problem was being let down by colleagues again!”

Doctor B: “Not the girlie wifey ones heading for part-time family doctor life?!” Doctor Doctor A: “Well, more like the ones just off the boat……”

*Is this form of conversation acceptable?*

**RELATED SCENARIOS**

1. A medical student who is not a regular drinker is convinced to join some medical colleagues for a night of drinking and clubbing to celebrate the end of term. The student has, at first, a pleasant evening, but eventually has a few drinks too many and ends up being escorted back to their accommodation by their friends. Once they have arrived, the student vomits profusely and responds to questions only by incoherent mumbling. The friends kindly agree to clean the drunk student up and put them to bed as they are covered in vomit. One of the medical students present takes several unflattering pictures of the drunk student in their messy state. A couple of weeks later, at a social, the medical student who took the photos lets slip the existence of the photos to some other students, and is egged on to post the photos on a ‘private’ medical student Facebook group.

1. A group of medical students create a group chat on Facebook which includes all student except one, an international student with limited English skills, which has been a barrier to integrating into the group. On the group chat, the excluded student is often ridiculed by a few of the others, however the international student is unaware of the existence of the group. One evening, a screenshot is made of a particularly amusing message on the group and shared with a non-medical student who is also friends with the international student, who feels compelled to show the ridiculing messages to them.

1. After a long day of placement in the Emergency Department, a medical student sees a news article on Twitter about a high-profile case of a terminally ill patient seeking legal means to end their own life. The student ‘tags’ you, their placement partner, in a reply to the link, saying “Wish they’d hurry up and legalise this, perhaps our beloved frequent flyers in the Emergency Department can give this a go and stop wasting our time!!”. You feel uncomfortable about this comment being posted in a public forum and suggest they delete the tweet, however the student tells you that you’re being a killjoy and nobody is going to take it seriously.

*Was it acceptable to take the photos in the first place? How about posting them on the group page? What are the potential consequences of such a photo being widely shared?*

*What could be the consequences of these events to a) the excluded student, and b) the perpetrators of the unkind messages?*

*Is it okay to make such comments in any setting? What about a private account? How do you think the following groups would react to seeing this: patients; journalists; academic/clinical supervisors?*

**3) Maintaining professional behavior in all aspects of life (issues: alcohol, lying, respect for colleagues, racism, competence/patient safety, sexual consent)**

# CLINICAL PRACTICE SCENARIO

Several junior doctors arrange a post-exam boat trip for their students and stock the boats with copious amounts of alcohol. Following a successful trip, during which no one falls in, they dock the boats and two of the doctors produce joints (cannabis) from their pockets, which they proceed to smoke and they offer it to the students, some of whom accept it. Later that evening, one of the junior doctors accompanies a rather drunk, female, first year medical student back to her accommodation. The next day the student confides in a fellow female first year medical student that she is concerned she may have had sex with the junior doctor, but cannot remember clearly what happened, except that he was with her when she awoke the following morning.

*What is the likely outcome for the junior doctor and what should he have done differently? If another student relates this story to you, what are your responsibilities?*

# RELATED SCENARIOS

1. After a weekend of heavy clubbing, considerable alcohol consumption, and little sleep, you awaken on Monday morning unable to bring yourself to go to your seminar. You know that you’ve already missed one or two, and might be near the boundary of ‘diligent attendance’, so you put a message on your seminar group WhatsApp chat asking someone to sign you in, promising that you’ll return the favour if anyone else needs it.

1. At the end of a practical class, anonymous questionnaires are handed out asking you to rate the practical element of the course and give any additional constructive feedback. You don’t see the point in these particular practical classes, so you give the lowest score possible in every category, and launch into a long, offensive, expletive-filled rant about how useless the class is. You also name some of the tutors who speak with a foreign accent asking for them to be replaced by people with accents that are easier to understand.

1. At Christmas, you are shadowing a hospital doctor and together you see a patient who requires an ECG to be taken and their blood sugar to be measured. The doctor asks if you have done these before which you say yes. The doctor asks you to do both procedures and walks off to make notes on a computer. You didn’t get a chance to say that you haven’t been trained in how to do these tasks in a clinical setting with patients. However, it’s a busy day, the doctor you are shadowing seemed relieved that he could leave you to do the tests, and you reason that, in theory, it can’t be too hard to extrapolate what you know to the clinical setting.

*Although it’s obviously acceptable to drink alcohol and go clubbing as a medical student, where is the line of professionalism? Is it okay to ask colleagues to sign you into one or two sessions? Is it fair to ask that of them? What if you were being asked to sign someone else in?*

*Is this okay? Would you appreciate feedback like this if you got involved in teaching? Do the comments about accents count as racism?*

*Should you go ahead? Is there any risk to the patient? How experienced do you need to be to carry out practical procedures on patients independently? Does it matter that you’re outside the University’s teaching and environment?*

**4) Health and probity (issues: alcohol/ addiction, lying, mental health)**

# CLINICAL PRACTICE SCENARIO

A family doctor is about to sit his final exams and is studying hard. He’s struggling to get on top of his studies and feeling extremely stressed, and, as a consequence, is having difficulty sleeping. One night he tries taking a pain killer tablet that was prescribed for his girlfriend several months ago and finds it really helps him sleep. He takes further pain killer tablets for the next 5 evenings but then her supply runs out.

The following day he is asked to perform a home visit for a patient with severe back pain. He takes a pack of pain killers from the practice dispensary anticipating that he may need to prescribe it to the patient to help with back muscles spasms. In the end the patient does not require pain killers, but he keeps the pack for himself, and continues to take pain killers frequently. His use escalates over the next few days so he’s taking it to help with symptoms of anxiety during the day as well as to help him sleep.

Over the next month he takes a further 2 packets of pain killers from the practice dispensary for “home visits”. On one occasion he is asked by the practice pharmacist to make sure he issues a prescription for any drugs he takes out with him, so he issues a prescription for one of his home visit patients, even though he does not offer them the medication. *What should this doctor have done differently?*

# RELATED SCENARIOS

1. After a night of heavy drinking and drug taking, a medical student had a row with her boyfriend. As she became angrier, she punched him in the face, then stabbed him in the abdomen with a bread knife and threw a laptop, a glass and a jam jar at him. He required hospital treatment but was discharged after 3 days.

1. One of the other medical students splits up with his girlfriend and spends Friday evening visibly distraught. On Saturday, he joins some students of other subjects on a pub crawl and becomes incredibly drunk. On the way back to medical school, he opens up to one of the other students about the situation with his ex-girlfriend and then becomes angry, suddenly punching a window, which he breaks. He is observed by a nearby police officer and is promptly arrested. His director of studies becomes aware the next day, after he is released from a cell overnight. Once sober, he is incredibly contrite, but very concerned about whether he will be allowed to qualify as a doctor.

1. Following a sporting back injury aged 17, a medical student struggles with chronic pain, worse after playing sports, which he has been advised not to play. He takes pain killers as required, in line with his family doctor’s instructions for the pain. However, during nights of heavy drinking, he gets a kick out of taking additional pain killer tablets and offering them to friends. You become aware of this practice and wonder what your responsibilities are.

*Is heavy drinking acceptable as a medical student or as a doctor? At what point is it not? What about illicit drug taking?*

*If caught doing something that puts one at the margins of fitness to practice, what is an appropriate way to respond?*

*If one is concerned that (i) oneself or (ii) a colleague is addicted to prescribed/ unprescribed legal or illicit drugs or has mental health issues, what is an appropriate course of action?*

# 5) Photos and communication (issues: Confidentiality and probity, respect for colleagues and patients, consent for use of publication of photographic material)

**CLINCAL PRACTICE SCENARIO**

A junior doctor is about to rotate to a new job and arranges to take a “team photo” of all the staff, including the nurses, ward clerk, physiotherapists and occupational therapist, with whom she has worked over the preceding 4 months. She proudly posts the photograph on Facebook with a message expressing her gratitude to them all. A week later, a patient writes an angry post on Facebook, asking for the photo to be deleted, as it contains her full name, date of birth and details of the operation she was about to undergo that day, which are written on a white board in the background. She also complains to the hospital directly and the deputy medical director contacts the junior doctor, who is now in a new post. The junior doctor immediately deletes the photo and then replies to the deputy medical director’s e-mail, saying that she has never posted any photos taken in the hospital on Facebook. She also comments that the patient in question was an “a mad old bat” and had clearly made the story up, as she probably wanted compensation from the hospital.

*What should this junior doctor have done differently?*

**RELATED SCENARIOS**

1. A final year student is meeting with his tutor and when asked by her how he will cope with long hour, working nights, untidy deaths and general anxiety about the job, he says he will communicate with his friends on WhatsApp as a way of coping. His tutor point out that patients can still be identifiable if they have rare conditions plus limited clinical details are sufficient to identify the patient. The student argues that WhatsApp is a secure interface, but his tutor points out that such information can only be shared between secure hospital based email accounts, as only then is information encrypted in transit.

1. A first-year medical student has a profile on an online dating app which allows them to view and chat with other users in the local area. Whilst taking part in interviews at a family practice a patient enters the room, and much to the surprise of the medical student, is a man with whom the student has ‘matched’ and conversed on their dating app, but has not met in person before. The student doesn’t know if the patient recognises them.

1. A second-year medical student meets a 19 year-old patient on the immunology ward. The patient has a rare condition which causes them to be severely immunocompromised, and as such has very limited freedom as they have to live in near-sterile conditions. The patient is excited to talk to someone their own age as most of the other patients are significantly older than them, and the patient’s friends are rarely able to visit. During the medical interview, the student develops a strong rapport and both parties enjoy the conversation. At the end of the interview, the patient is clearly disheartened that the student has to leave. A few hours later, the student receives a friend request from the patient on Facebook, and a message saying how much they enjoyed the visit, and that they should continue chatting online.

*How can you ensure that any communications maintain professional boundaries and confidentiality?*

*What should be done in this situation? Would it be appropriate to continue with the interview, and if not, why? (No case-specific guidance in the document linked below)*

*Is it okay to accept the friend request in this situation? Is it ever okay to start a non-professional relationship (platonic or romantic) with current or former patients?*

**6) Presentation and conduct (issues: appearance, sphere of competence, data protection)**

# CLINICAL PRACTICE SCENARIO

A patient who was admitted on Thursday with chest pain suddenly deteriorates and develops further chest pain and becomes breathless. The junior doctor is unsure what to do and calls the on-call attending cardiologist on his mobile.

The attending states he’s not by a computer and asks the junior doctor to text him a picture of the patient’s ECG. He then calls back advising that the patient need to be transferred to a specialist centre. The junior doctor attempts to explain this to the patient and his family, but they become distressed and ask to speak to the attending. The junior doctor calls the attending and asks if he can come to the ward. He seems somewhat reluctant. About 60 mins later her arrives and is dressed casually in shorts and sandals. He smells slightly of alcohol but is not drunk and explains he was at a BBQ. He declines to speak to the family himself but gives a further explanation to the junior doctor about what the ECG shows and makes a brief call to the specialist centre himself. He then leaves again after 15 mins declining to answer any further questions.

*What should these doctors have done differently? Are they operating within their spheres of competence?*

# RELATED SCENARIOS

1. Your tutor notices that you are wearing trainers whilst on the ward, and despite the rest of your clothing being professional, instructs you to go home to change into more suitable footwear. You argue that it’s impractical to cycle to teaching sessions in ‘work shoes’ and that the ward is quite far away from your house. However, the tutor insists; although you’d rather start straight away and see some patients, you do as asked and return later.

1. When you return, you find out that you will be interviewing a patient at their home. The patient you interview tells you that in the last couple of days they’ve been worried as they’ve experienced new, intermittent chest pain when walking up stairs at home. You conclude that the ‘chest pain’ is probably just indigestion, reassure the patient that there’s probably nothing serious happening, and that taking some antacid tablets is probably the best course of treatment. The patient seems relieved.

1. Back at medical school you scan the notes you made during your interview. However, you accidentally leave a page of your notes in the scanner. The next day, you realise the page is missing, and that you had written the patient’s name and address on the page so you could find their house; you had written personal details of the patient’s long-term health condition on the page. You return to the scanner, but the paper has gone, and you can’t find it in anywhere in the room. It seems likely to you that another student removed the paper and put it in the bin, which was probably then emptied.

*Was the tutor right to send you back to change your footwear? Why is it important to maintain a professional appearance when meeting patients?*

*What harm could potentially come to the patient because of your reassurance? What steps should be taken in such a situation? Is there any level of medical advice that it’s appropriate for you to give at this stage in your training?*

*What action should be taken now, if any? What are the potential consequences of patient ‘data loss’?*
