# Supplementary material for: Using a Scenario-Based Approach to Teaching Professionalism to Medical Students: Course Description and Evaluation
Source: JMIR Med Educ. 2021 Jun 24;7(2):e26667. doi: 10.2196/26667 (PMC8277325; doi:10.2196/26667)
Supplement: Multimedia Appendix 2 [file mededu_v7i2e26667_app2.docx]

*Entry to the Profession: A Scenario-based Approach to Teaching Professionalism to* *Medical Students*

**Background**

Medical education involves not only the accumulation of factual and practical knowledge. A medical student must also gain a clear understanding of professional behaviour and this behaviour must apply not only in the workplace or other university/ hospital setting, but in other aspects of life. Such professional values are clearly laid out in the General Medical Council (GMC) documents “Tomorrow’s doctors” [1] and “Good Medical Practice”[2]. Those of us involved in this project have encountered a wide range of problems with medical students at college and university level including violent crime against people and property while drunk, illicit drug taking, prescribed drug addiction, suspected alcohol dependency, inadvertent pregnancy due to binge drinking, theft and undiagnosed/ untreated mental health problems. While further education about professional behaviour may not prevent all of these, it is likely to lower the frequency of many of them, particularly if delivered early in the course and in a context of peer-based education, where more considered behaviour becomes the norm. Education is also usually more effective if it is active and delivered in a supportive, relaxed environment, where students can discuss their views openly.

**Aim**

To provide students with an understanding of the professional responsibility they have in all aspects of their conduct from their time as a first year medical student onwards.

**Approach**

A small group session which is deliberate to make the sessions non-threatening, in groups where students will know each other well and are more likely to contribute and to feel that their peer group will strive to maintain professional behaviour, is essential. In each session, we will consider 3 examples of poor professional conduct by doctors, (drawn either from personal experience of medicolegal and medical board expert practice). The poor professional conduct of the doctor will be related to 3 scenarios showing analogous behaviour which students can relate to more directly, in order to stimulate discussion about what might and might not be appropriate behaviour while a medical student.

On each occasion, 3 scenarios will be covered, and the total group of students will be divided into 2 or 3 groups, with each group discussing one of the scenarios and then feeding back to the group as a whole. The remainder of the group will then have the opportunity to ask questions. If there are only 2 groups discussing scenarios, the lead facilitator will discuss the third set of scenarios with the whole group of students towards the end of the session. Supervisors/ tutors at any participating college will be provided with a vignette for each scenario and then key discussion points that they should ensure are raised at some point in the discussion, with clear links to GMC guidance and other relevant documents. We suggest using scenarios similar to the provided case vignette 1 – 3 in the first session and 4 – 6 in the second.

A reasonably senior clinically qualified preclinical director of medical studies or supervisor should oversee the sessions, assisted by other interested clinically qualified colleagues and/or clinical students with patient experience undertaking this as part of their learning to teach a course. We would very strongly suggest that you provide the students with paper copies of this material only, as electronic copies could be taken substantially out of context or could cause reputational damage to an institute if that occurred.

**Issues to be addressed by individualised scenarios**

Confidentiality

Social media

Respect for colleagues

Stealing / lying

Violence to person / property

Alcohol and drugs

Prescription drugs

Racism / sexism

Sexual consent

Mental health - of self and others

Academic bullying

Whistle blowing / covering up

Team-working

Behaviour / clothing

**Expanding the session**

Roll out of sessions need to be undertaken in small groups of students who know each other reasonably well (i.e., with a structure analogous to the described study), to make the environment non-threatening and conducive to discussion. Carefully structured and individualised questionnaires, anonymised, to test understanding of and attitudes towards professional behaviour, as a function of whether or not they have been exposed to the

professionalism sessions will be essential.

1. http://www.gmc-uk.org/Tomorrow_s_Doctors_1214.pdf_48905759.pdf

2. http://www.gmc-uk.org/static/documents/content/GMP_.pdf
